# Epidemiologic Features and Risk Factors of Crimean–Congo Hemorrhagic Fever in Samsun Province, Turkey

**DOI:** 10.2188/jea.JE20120097

**Published:** 2013-03-05

**Authors:** Aziz Sisman

**Affiliations:** Ondokuz Mayis University, Faculty of Engineering, Department of Geomatics, Samsun, Turkey

**Keywords:** Crimean-Congo hemorrhagic fever, epidemiology, geographical information systems, Turkey

## Abstract

**Background:**

Crimean–Congo hemorrhagic fever (CCHF) is a tick-borne infectious disease that has a considerable mortality risk and is a challenge for the population of endemic rural areas and health care workers. This study investigated the epidemiologic features and main risk factors of CCHF in Samsun Province, Turkey, using CCHF cases diagnosed from 2007 to 2011 recorded by the Samsun Provincial Health Directorate.

**Methods:**

In the study area, 126 cases were evaluated statistically and spatially. Minitab 16 software was used for statistical analysis of the data, and ArcGIS 9.3 software was used for spatial analysis.

**Results:**

Among those who received a diagnosis of CCHF, 69 (54.7%) were male, 57 (45.3%) were female, 114 (90.5%) were discharged, and 12 (9.5%) died. A total of 112 of the 126 (88.9%) cases occurred at an altitude higher than 600 m. In addition, 84.1% of cases were reported during May through July, which are the busiest months for those working in the agriculture and animal husbandry sectors.

**Conclusions:**

CCHF causes severe disease and has a high mortality rate (about 10% in Turkey). Early diagnosis of CCHF can be improved by periodic education of people at high risk, ie, men and women working in agriculture and animal husbandry in rural areas and those working in health care.

## INTRODUCTION

The Crimean–Congo hemorrhagic fever (CCHF) virus is part of the *Bunyaviridae* family and belongs to the genus *Nairovirus*.^1^ CCHF is a zoonotic disease that causes severe hemorrhagic fever in humans after infection via tick bites or direct contact with body fluids or tissues of viremic patients or viremic livestock.^2^^–^^6^ After an incubation period of 2 to 7 days, the patient has flu-like symptoms that rapidly progress to a hemorrhagic state with bleeding from the mucous membranes and petechiae, often associated with thrombocytopenia and leukopenia. CCHF has a high case-fatality rate, from 10% to 60%, which makes it a public health concern.^1^^,^^7^^,^^8^

CCHF was first described during World War II, in the Crimean region of Russia. The virus responsible for Crimean hemorrhagic fever was later shown to be antigenically identical to the Congo virus, which was isolated from a febrile patient in the Congo in 1956. The virus has since been referred to as Crimean–Congo hemorrhagic fever virus.^9^^,^^10^ CCHF has a very wide geographic distribution, as it is endemic in more than 30 countries in Africa, central and southwestern Asia, the Middle East, and southeastern Europe.^10^ During the last decade, climatic, environmental, and anthropogenic factors have driven the expansion of CCHF endemic foci and the onset of community outbreaks.^11^

The risks of tick attachment and related tick-borne diseases in a given region are related to vector tick abundance and human activities. Also, geographic and climatic conditions and the presence and habitat preferences of host animals are closely related with the risk factors.^12^ In Turkey, the first cases of CCHF virus infection were reported in 2002, and since then the number of cases has gradually increased.^10^^,^^13^ By 2009, the total number of CCHF cases reached 4453, and the total number of patient deaths was 218.^14^ CCHF infection is also an important public health issue in Turkey because of its high case-fatality rate.^15^ Most cases have been reported in the central Black Sea region and central Anatolia.^8^ This increase in Turkey may be attributed to its location on the migratory path between Africa and Europe and to agricultural activities, environmental factors, and changes in climatic factors.^6^^,^^10^^,^^11^ Turkey’s geography and climate provide a suitable environment for ticks,^10^ which enjoy a wide host spectrum in Turkey, ranging from small rodents to wild animals and from birds to household pet mammals. Approximately 32 species of tick have been reported.^3^^,^^10^^,^^16^^,^^17^ The genus *Hyalomma* is frequently seen in the Black Sea region, perhaps because this region is rainy and densely forested.^13^^,^^18^ The habitats and migratory paths of wild birds, the 2 most important of which are found in Samsun, are also important in the spread and settlement of CCHF in the region.^10^^,^^13^

Geographic information systems (GIS) have been increasingly used in studies of the population dynamics of various arthropod vectors in relation to a range of ecologic factors and disease prediction.^19^^,^^20^ GIS allow investigation of a number of factors, including the monitoring of the development and spread of diseases in a particular area, evaluation of risk factors, development of control strategies for health incidents, and the management and planning of health services. Due to recent advances in GIS technologies and statistical techniques, health and population data in a geographically defined region can be analyzed simultaneously; thus, the systematic investigation of geographical variations in disease risks is possible, and the epidemiologic progress of diseases can be better understood.

The aims of this study were (1) to determine the epidemiologic features (including age, sex, location, fatality, and endemic area) of CCHF cases identified at hospitals in Samsun province from 2007 through 2011 and (2) to produce a case distribution map that shows the epidemiologic risk area for CCHF using GIS.

## METHODS

**Figure 1.**
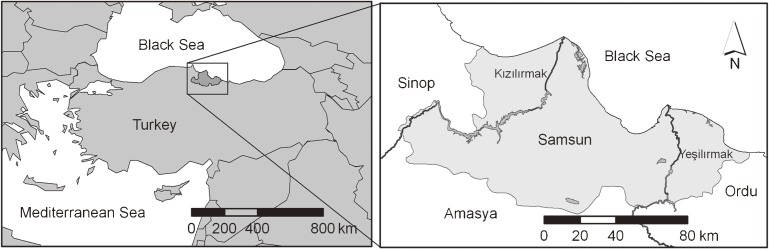
Study area

This study was done after obtaining the necessary legal permission from the Samsun Provincial Health Directorate. Cases of CCHF diagnosed at hospitals in Samsun province between 1 January 2007 and 31 December 2011 and recorded by the Samsun Provincial Health Directorate were studied. Samsun is a coastal province in the central Black Sea region of Turkey. It lies between 40° 85′ and 41° 75′ North and between 34° 90′ and 37° 20′ East (Figure 1), covers an area of about 9083 km^2^, and has a population of about 1 250 000. The climate is generally moderate; however, the climates of the coastal strip and interior regions differ. The moderating influence of the Black Sea climate can be seen along the coastal strip: summers are hot and winters are warm and rainy. In the interior regions, which are influenced by the 2000-meter-high Akdağ Mountain and 1500-meter-high Canik Mountain, summers are cool and winters are cold, rainy, and snowy.^21^

Samsun Province has a rich and fertile agricultural area, and there are 2 important plains—Bafra and Çarşamba—which are located around the Kızılırmak and Yeşilırmak rivers. In Samsun Province, agriculture and livestock workers are present from the seaside (Bafra plain and Çarşamba plain) to the high plateaus. Sheep and cattle are raised in Samsun, which increases possible close contact of humans with ticks and animals. This situation is also seen in other central Black Sea provinces^22^ such as Tokat, Amasya, and Çorum.

In the current study, data from 138 cases of CCHF diagnosed between 1 January 2007 and 31 December 2011 were obtained from the Samsun Provincial Health Directorate. The address data for the 126 patients were supplied solely in the form of province, district, and village name. After analysis of the age, sex, and address data of the 126 cases, disease incidences were calculated, and box-and-whisker plots were prepared. The box is the area between the 25th and 75th percentile values. The whisker is the perpendicular line extending through the box. Observed values that fall along the length of the whisker are considered to not significantly differ from the median. Minitab 16 software was used for statistical analysis of the data. Then, the provincial boundaries, district boundaries, rivers, and lakes of Samsun Province were digitized and entered into the ArcGIS 9.3 software. The coordinates and altitudes for the 126 CCHF cases whose full addresses had been determined were established from standard topographic maps (scale 1:25 000) and transferred to a digital map. Different variables were examined using spatial and nonspatial data entered into the ArcGIS software.

## RESULTS

The first case recorded in Samsun by the Samsun Provincial Health Directorate was in 2004. From 1 January 2007 to 31 December 2011 in the Samsun provincial state hospitals and Ondokuz Mayıs University Hospital, 138 patients from 14 districts of Samsun province received a diagnosis of CCHF. In the current study, address data for 126 (91.3%) cases were supplied, 114 (90.5%) of whom were concentrated in 5 districts: Havza, Ladik, Kavak, Vezirköprü, and Asarcık.

Of the patients diagnosed with CCHF, 69 (54.7%) were male and 57 (45.3%) female, and the median age of patients was 47.5 (31.7–59.0) years. The mean age of men and women was 48.0 (36.0–60.0) and 44.0 (30.5–57.0) years, respectively; the difference was not statistically significant. Address data showed that 118 (93.7%) patients lived in rural areas, as workers in agriculture and animal husbandry. Selected demographic characteristics and district of residence of patients, by year, are shown in Table 1. Three (2.38%) of the patients were health care workers who acquired the disease via contact with infected blood; 1 of these 3 health care workers died, in 2009.

**Table 1.** Selected demographic characteristics of cases by year, No. (%)

		2007	2008	2009	2010	2011
No. of cases		4	39	35	25	23
No. discharged		4	37	31	21	21
Deaths		0 (0)	2 (5.1)	4 (11.4)	4 (16.0)	2 (8.7)

Sex	Male	3 (75.0)	23 (59.0)	14 (40.0)	15 (60.0)	14 (60.9)
	Median age	40	45	52	55	51.5
	Female	1 (25.0)	16 (41.0)	21 (60.0)	10 (40.0)	9 (39.1)
	Median age	7	34.5	48	39.5	62

District	Asarcık	0	3 (7.7)	0	3 (12.0)	0
	Havza	2 (50.0)	12 (30.8)	13 (37.1)	6 (24.0)	5 (21.7)
	Kavak	0	2 (5.1)	11 (31.4)	2 (8.0)	9 (39.1)
	Ladik	0	7 (17.9)	5 (14.3)	10 (40.0)	8 (34.8)
	Vezirköprü	0	7 (17.9)	5 (14.3)	3 (12.0)	1 (4.3)
	Others	2 (50.0)	8 (20.6)	1 (2.9)	1 (4.0)	0

**Figure 2.**
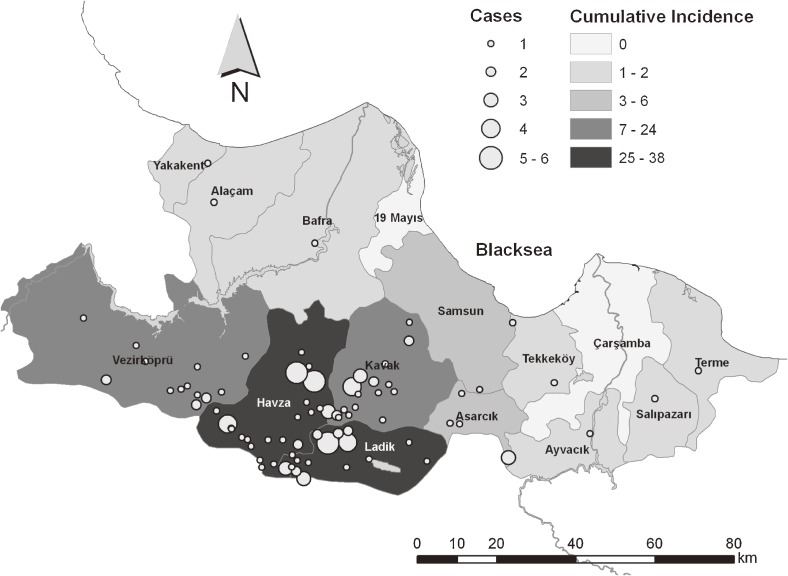
Cumulative incidence and distribution of cases by district

In the study area, 114 (90.5%) CCHF patients were discharged and 12 (9.5%) died. The CCHF fatality rate was 9.5 in the study area during 2007–2011. The cumulative incidence and district distribution of cases were calculated for 2007–2011, as shown in Table 2 and Figure 2. The cases were concentrated in 5 districts located in south Samsun Province.

**Table 2.** Incidence of Crimean–Congo hemorrhagic fever by district, 2007–2011

District	Population 2010	Total cases	Incidence (per 10 000)	Total deaths	Altitude of district center
Asarcık	18 867	6	3.18	1	790
Havza	45 188	38	8.41	3	675
Kavak	20 940	24	11.46	3	600
Ladik	17 849	30	16.81	1	930
Vezirköprü	105 447	16	1.52	3	335
Others	1 045 795	12	0.11	1^b^	29.4^a^

^a^Average altitude of other districts.

^b^Medical Staff in Faculty of Medicine.

As shown in Figure 2 and Table 2, 114 (90.48%) cases were recorded in 5 districts. These districts have large areas devoted to agriculture and animal husbandry, as in other districts of Samsun. However, these 5 districts substantially differ from other districts in ambient temperature and altitude. The 5 districts are located in south Samsun Province. The climate is drier, and the temperature difference between day and night is greater, than in the other districts of Samsun. The long-term average temperature of these districts during the season of high tick activity was 16.1°C.^23^ The other districts of Samsun Province are under the moderating influence of the Black Sea climate, and the long-term average temperature in these districts during the season of high tick activity was 18.8°C.^23^

**Figure 3.**
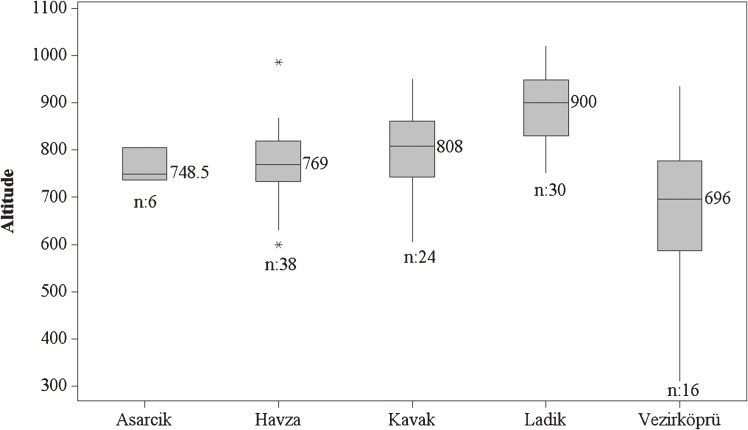
Box plots of altitude of patient homes by district

Investigation of the altitudes at which the cases were reported revealed that 119 cases (94.4%) occurred at an altitude higher than 300 m and 112 cases (88.9%) occurred at an altitude higher than 600 m. These findings suggest that altitude is an important factor in CCHF incidence. The box-and-whisker plot in Figure 3 shows the distribution of CCHF cases that occurred at an altitude higher than 300 m. This box plot shows the minimum value, 25th percentile, median, 75th percentile, maximum value, and outliers. The box represents the interquartile range of the data set. The dimensions of the boxes indicate that 50% of cases occurred at an altitude of 600 to 800 m in Vezirköprü. This proportion is higher than that of the other districts. There are 2 outliers in the Havza District, and the median of the Ladik district indicates that 50% of cases occurred below an altitude of 900 m. This value is considerably higher than in other districts.

**Figure 4.**
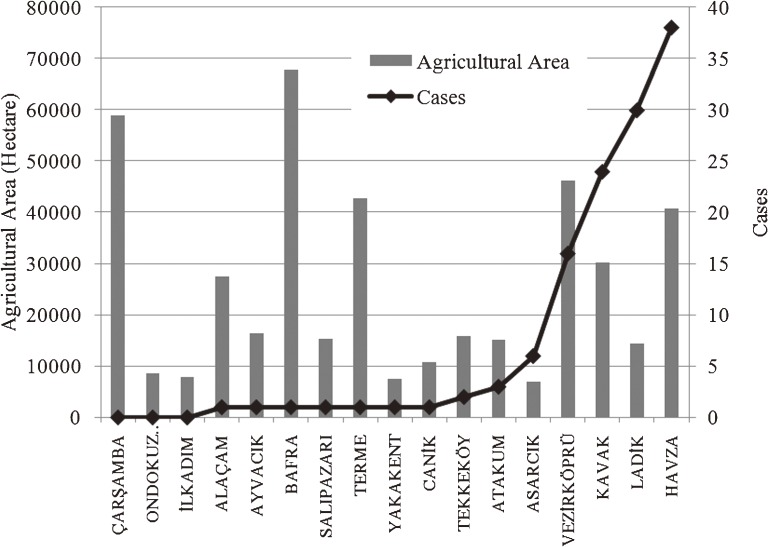
Total agricultural area and number of cases by district

**Figure 5.**
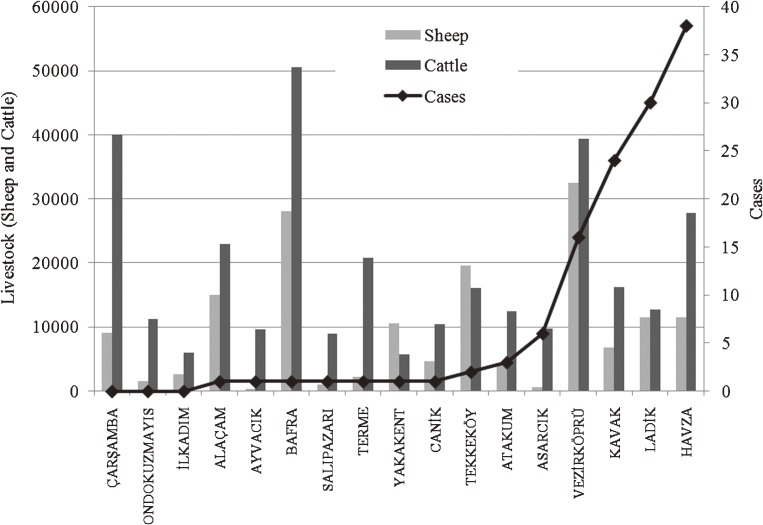
Total number of livestock and number of cases by district

**Figure 6.**
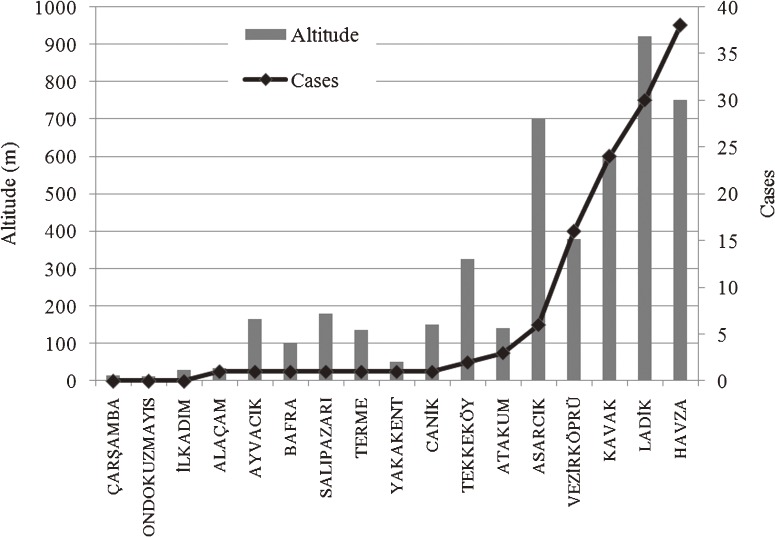
Relationship between altitude of patient homes and number of cases by district

A total of 118 (93.7%) CCHF cases were individuals living in rural areas (working in agriculture and animal husbandry), 114 (90.5%) of which were concentrated in 5 districts: Havza, Ladik, Kavak, Vezirköprü, and Asarcık. Figure 4 shows the relationship between total agricultural area and number of cases.^24^ Although the Bafra and Çarşamba districts have the largest agricultural area, the number of cases is lower than in most other districts. Figure 5 shows the relationship between total number of livestock (cattle and sheep) and number of cases,^25^ and Figure 6 shows the relationship between altitude and number of cases, by district, in the study area.

**Figure 7.**
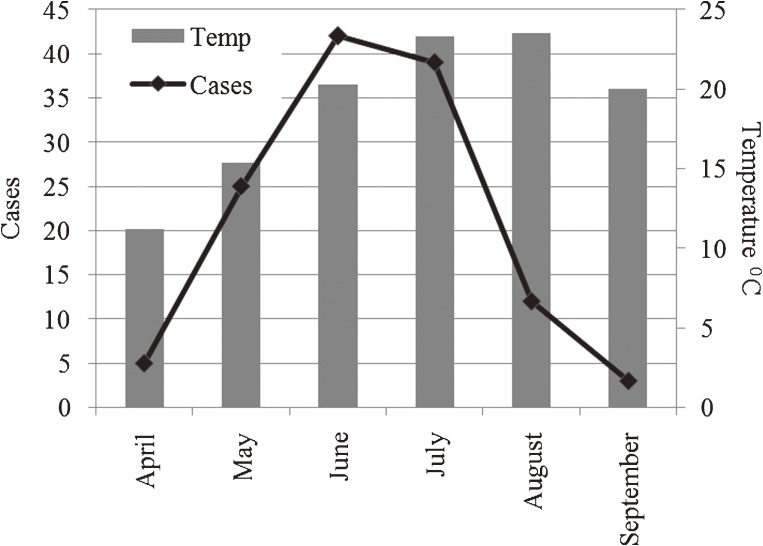
Relationship between number of cases and temperature

Tick activity increases with rising temperature. Because Turkey is in the Northern Hemisphere, temperatures start to rise in the spring and the virus carried by the ticks is most active from June through September.^26^^,^^27^ Samsun Province has a generally moderate climate; temperature starts to rise from April. In the current study, 106 of the cases (84.13%) were reported from May through July, which are the busiest months for those working in the agriculture and animal husbandry sectors. Twelve (9.52%) of the cases were reported in August, and only 5 (3.97%) were reported in April in this study. Figure 7 shows the relationship between monthly number of cases and temperature, from April through September. The monthly distribution of cases and average temperature, by district, are shown in Table 3.

**Table 3.** Number of cases and average temperature^23^ by month^a^ and district

District	Cases (°C)					

	April	May	June	July	August	September
Asarcık	0 (10.5)	2 (13.1)	4 (16.5)	0 (18.7)	0 (19.7)	0 (16.3)
Havza	0 (10.1)	9 (13.4)	10 (17.0)	17 (19.1)	1 (19.4)	1 (15.8)
Kavak	1 (10.5)	2 (13.1)	9 (16.5)	8 (18.7)	3 (19.7)	1 (16.3)
Ladik	3 (8.9)	10 (12.5)	7 (15.6)	4 (17.7)	6 (17.6)	0 (14.4)
Vezirköprü	1 (12.2)	1 (16.0)	7 (19.7)	6 (22.1)	1 (22.5)	0 (18.9)
Others	0 (11.2)	1 (15.4)	5 (20.3)	4 (23.2)	1 (23.2)	1 (19.5)

^a^Months in which cases were recorded.

## DISCUSSION

Although the first cases were concentrated around the Kelkit Valley, other cases were later reported in 27 provinces,^21^^,^^28^ including Samsun. Four cases were reported in Samsun in 2007, and the number rose to 38 in 2008. Studies have investigated the potential roles of migratory birds and movement of livestock carrying ticks in the spread of the virus over distant geographic areas.^10^ It has been suggested that birds migrating from the Balkans caused the 2002 CCHF outbreak in Turkey.^28^

Field studies begun in Turkey in 2005 mapped the area inhabited by *Hyalomma marginatum*, which is linked to CCHF epidemics, and identified potential risk areas for the disease. Disease risk was closely correlated with mixed land-use structures such as forests, flat pasturage, grazing land, pastureland, and bush, and with vector tick density.^28^^,^^29^ Although these studies added to the base of specific knowledge on the epidemiology of CCHF, the epidemiologic factors underlying the spread of CCHF in Turkey were not fully clarified, which gave rise to a need for further research on the subject.

This study attempted to determine the epidemiologic features of CCHF diagnosed in Samsun, which extends from sea level to 2000-m mountain peaks. The median age of patients was 47.5 years and 54.7% were men and 45.3% were women. These nearly equal proportions of men and women reflect the fact that approximately equal numbers of men and women work in agriculture and animal husbandry in rural areas of Turkey. Both these occupational categories are at risk of tick contact and thus CCHF.^4^^,^^16^^,^^30^^,^^31^ Epidemiologic studies have reported that CCHF is also more frequent in regions with forests and pasturages. In addition, living in villages or rural areas has been described as a risk factor.^32^^,^^33^

Few studies have examined the altitude at which people contracted CCHF. Zivalioglu^26^ reported that around 74% of people with CCHF lived between 600 and 1200 m above sea level (average, 800 m). We suspect that in Turkey, in other countries in which CCHF occurs, and particularly in the study region, the distribution of ticks carrying the disease is denser at high altitudes and that there is a correlation with animal husbandry, which frequently takes place at such elevations.^28^^,^^29^ In fact, 94.4% of the present patients lived at an altitude of 300 m or higher and 84.9% lived at an altitude between 600 and 950 m.

Tick activity and CCHF risk increase with rising temperature in spring.^26^^,^^27^ In the study area, 106 (84.13%) cases were reported from May through July, which are the busiest months for work in agriculture and animal husbandry. There was a strong correlation between CCHF risk and animal husbandry, agricultural activities, altitude, and season. However, animal husbandry and agricultural activities were not associated with individual risk in the study area, as shown in Figures 4 and 5. Disease risk is associated with animal husbandry and agriculture activities from May through July in arid areas at altitudes between 600 and 950 m.

The widespread geographic distribution of the CCHF virus in Turkey and globally, together with its severe impact on human beings, makes CCHF a public health concern. The average case-fatality rate is 30% to 50%; however, case-fatality rates of 10% to 80% have been reported in some outbreaks.^1^^,^^7^^,^^8^^,^^34^ The case-fatality rate is usually higher for nosocomial infections than after tick bites, possibly due to a difference in virus dose. Geographic location also seems to influence death rate. Particularly high case-fatality rates have been reported in some outbreaks in the United Arab Emirates (73%) and China (80%).^35^ In the current study, 126 cases were identified, and 12 patients died from CCHF infection during 2007–2011. The fatality rate was between 0% and 16% and has started to decrease since 2010. However, the lower case-fatality rate may also be influenced by the fact that rigorous supportive treatment is available in hospitals where most CCHF cases occur. This improved care is linked to the expertise Turkey has gained in CCHF treatment.

### Conclusion

CCHF causes severe disease and has a mortality risk of about 10% in Turkey. High-risk groups are men and women working in agriculture and animal husbandry in rural areas, especially those living at an altitude of 600 m or higher, during the months of May, June, and July. Health care workers (particularly in infection services) also have a higher risk of CCHF. Although Turkey has gained expertise in treating CCHF, clinicians should be regularly updated on the virus. Furthermore, periodic education of people in high-risk groups is important for early diagnosis and in preventing the virus from becoming established in new areas, which may lead to subsequent outbreaks. These measures will help contain and eliminate CCHF in areas where it is endemic.
